# Supplementary material for: Margin Free Resection Achieves Excellent Long Term Outcomes in Parathyroid Cancer
Source: Cancers (Basel). 2022 Dec 29;15(1):199. doi: 10.3390/cancers15010199 (PMC9818355; doi:10.3390/cancers15010199)
Supplement: Supplementary file 1 [file cancers-15-00199-s001.zip › cancers-2049110-Supplementary Table S2.pdf]

Supplementary Table S2: Outcome in single centre series of parathyroid cancer											
**Year**	**Lead author**	**ref**	**type**	**n**	**Follow-up -at 5 years -median/mean**	**OS 5-year**	**RFS 5-year**	**DSS 5-year**	**OS 10-year[a]**	**RFS 10-year[b]**	**DSS 10-year**
1992	Wynne	[28]	single	43	43 mean: 6.9y	69%	-	-	-	-	-
1998	Chow	[29]	single	10	5	5/5 100%	4/5 80%	4/5 80%	4/4 100%	-	-
2001	Dotzenrath	[30]	single	4	2	2/2 100%	0/2	2/2 100%	2/2 100%	0/2	2/2 100%
2003	Munson	[31]	single	61	57	44/57 76.9%	32/57 56.6%	47/57 82.0%	-	-	-
2004	Busaidy	[32]	single	27	24	19/24 79.2%	14/24 58.3%	20/24 83.3%	8/17 47.1%	5/17 29.4%	12/17 70.6%
2007	Iihara	[33]	single	38	38	35/38 92.1%	28/38 73.7%	35/38 92.1%	34/38 89.5	12/38 72.2%	34/38 72.2%
2011	Harari	[9]	single	37		78.3%			66.7%		
2012	Karakas	[34]	single	19	19	18/19 93%	7/19 36.8%		8/19 43%		
2015	Basceken	[35]	single	11	9	8/9 88.9%	8/9 88.9%	8/9 88.9%	5/6 83.3%	5/6 83.3%	5/6 83.3%
2016	Xue	[36]	single	40	5y: n.a. median: 4.1y	78.9%			60.7%		
2017	Silva-Figueroa	[38]	single	68	68	54/68 80%	42/68 62%	n.a.	46/68 68%	35/68 51%	n.a.
2017	LIBÁNSKÝ	[37]	single	16	16	16/16 100%	12/16 75%	16/16 100%	-	-	-
2019	Asare	[39]	single	27	5y: n.a. median: 6.4y	78%			61%		
2020	Zheng	[40]	single	7	7	6/7 85.7%	6/7 85.7%	7/7 100%	-	-	-
2021	De Pasquale	[41]	single	8	5y: n.a. mean: 3.2y	n.a.	-	-	-	-	-
2021	Sali	[42]	single	20	5y:n.a. median: 21.5	n.a.			n.a.		
2022	Cunha	[43]	single	17	5y: 64.7%	8/11 72.7%	4/12 33.2%	10/11 90.9%	8/10 80.0%	3/10 30.0%	8/9 88.9%
2022	Wei	[44]	single	31	31	50%	-	-	-	-	-
	**all**					**50.0-100%**	**33.2-88.9%**	**80.0-100%**	**43.0-100%**	**29.4-83.0%**	**72.0-100%**
	**median**					**79.2%**	**62.0%**	**90.9%**	**68.0%**	**51.0%**	**83.3%**
**2022**	**Schulte**		**single**	**25**	**24**	**23/24 95.8%**	**19/23 82.6%**	**19/19 100%**	**16/19 84.2%**	**15/19 79.0%**	**23/23 100%**

a Denominator: n patients who died of any cause or have a follow-up of 10 years; numerator: n patients alive patients at FU of at least 120 months

b Denominator: n patients who died of any cause or have a follow-up of 10 years; numerator: n patients D/other or alive at FU of at least 120 months without recurrence
