# Supplementary material for: Margin Free Resection Achieves Excellent Long Term Outcomes in Parathyroid Cancer
Source: Cancers (Basel). 2022 Dec 29;15(1):199. doi: 10.3390/cancers15010199 (PMC9818355; doi:10.3390/cancers15010199)
Supplement: Supplementary file 1 [file cancers-15-00199-s001.zip › cancers-2049110-Supplementary_Table_S1_Patient cohort.pdf]

Supplementary Table S1: Patient cohort - treatment and outcome data

patient	gender	age at diagnosis	year of 1st op	initial surgery	RLN resection	resection margin	adjuvant therapy	local recurrence after 1st surgery	time to local recurrence	Year of 2nd op	local recurrence after 2st surgery	adjuvant therapy	distant metastases	time to distant metastases	death due to disease	overall follow-up (months)	histology	level VI (+/total)	level II-V (+/total)
15	F	63	1996	LE	No	R1	no	No	--	--	--	no	No	--	No	318	LR	nd	nd
5	F	62	2005	EB	No	R0	no	no	--	--	--	no	no	--	no	202	HR	0/2	nd
7	F	67	2006	EB	No	R0	no	no	--	--	--	no	no	--	no	185	HR	0/1	nd
2	M	66	2006	EB	Yes	R1	no	Yes	13	2009	--	RT (55Gy) 20#	lung	58	Yes	130	HR	2/31	dc
1	F	57	2007	EB	No	R0	no	no	--	--	--	no	no	--	no	150	HR	0/8	nd
4	F	52	2007	EB	No	R0	no	no	--	--	--	no	no	--	no	130	LR	0/16	nd
9	F	47	2008	EB	No	R0	no	no	--	--	--	no	no	--	no	172	HR	0/7	0/12
8	M	33	2008	EB	No	R0	no	no	--	--	--	no	no	--	no	171	LR	0/3	nd
3	M	40	2008	EB	No	R0	no	no	--		--	no	no	--	no	134	LR	0/3	0/43
10	F	63	2009	EB	Yes	R0	no	no	--		--	no	no	--	no	128	LR	0/9	0/23
17[a]	M	57	2010	LE	No	R1	no	Yes	29	2013	No	RT (60 Gy) 30#	No	--	No	137	HR	nd	nd
11	M	37	2010	EB	Yes	R0	no	no	--	--	--	no	no	--	no	120	HR	0/10	nd
18[a]	M	81	2010	LE	No	R1	no	Yes	Persistent	2012	No	RT (60 Gy) 30#	No	--	No	83[b]	HR	nd	nd
12	F	75	2011	LE	No	R1	RT (60 Gy) 30#	no	--	--	--	no	no	--	no	123[b]	LR	nd	nd
16[a]	F	63	2011	LE	No	R0	no	No	--	--	--	no	No	--	No	134	LR	nd	nd
19[a]	F	64	2011	LE	No	R1	no	Yes	14	2013	No	no	No	--	No	127	LR	nd	nd
13	F	54	2012	EB	No	R1	RT (60 Gy) 30#	no	--	--	--	no	no	--	no	117	LR	0/3	0/32
14	F	58	2013	EB	Yes	R0	no	no	--	--	--	no	no	--	no	104	HR	0/15	nd
6	F	59	2013	EB	No	R0	no	no	--	--	--	no	no	--	no	63[b]	LR	0/3	nd
21	F	81	2013	EB	No	R0	no	No	--	--	--	No	No	--	No	14[b]	LR	0/5	nd
22	M	41	2014	LE	No	R0	no	No	--	--	--	No	No	--	No	95	HR	0/28	nd
23	F	43	2014	EB	No	R1	no	No	--	--	--	No	No	--	No	82	HR	nd	nd
20	F	46	2015	EB	No	R0	no	No	--	--	--	No	No	--	No	75	LR	nd	nd
24	F	82	2016	LE	No	R0	no	No	--	--	--	No	No	--	No	61	HR	0/5	nd
25	M	71	2018	EB	No	R0	no	No	--	--	--	No	No	--	No	49	LR	0/12	nd

High Risk (HR)
Low Risk (LR)
nd: lymph node dissection not done
(+/total): positive lymph node on histology over total excised
en bloc (EB), Local excision (LE), Recurrent laryngeal nerve (RLN)
RT: radiotherapy
#: fractions
Patient 15 was operated on in 1996 by another surgeon at the centre.
Patient 12, 22, 24 were not suspected to have cancer and underwent local excision alone
[a]: Patient had initial surgery elsewhere and were operated for recurrence at KCH
[b]: Patient died due to other causes
