# Supplementary figures and images for: Margin Free Resection Achieves Excellent Long Term Outcomes in Parathyroid Cancer
Source: Cancers (Basel). 2022 Dec 29;15(1):199. doi: 10.3390/cancers15010199 (PMC9818355; doi:10.3390/cancers15010199)

Supplementary Figure S1: PRISMA Flow Diagram

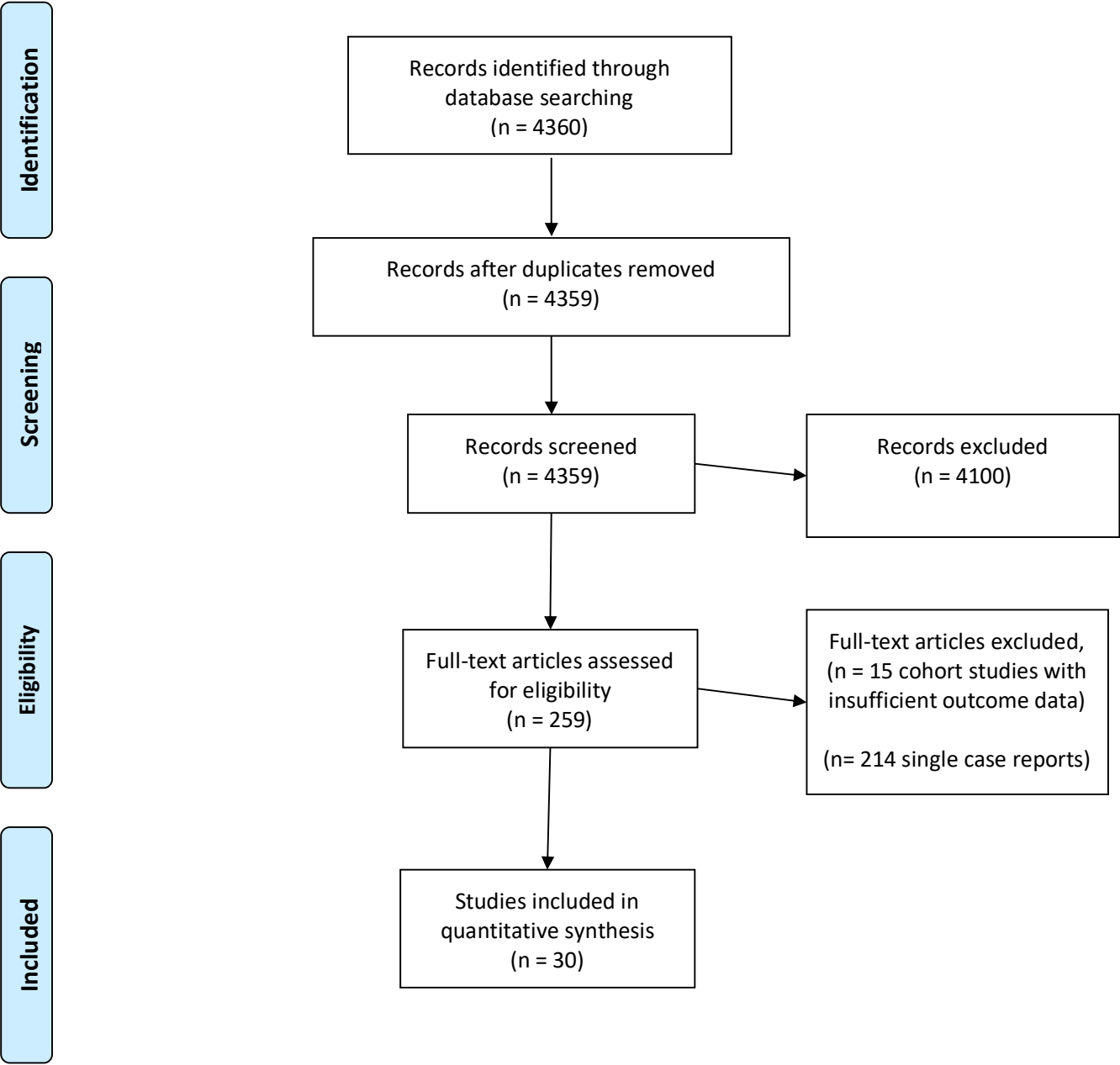

Supplement: Supplementary file 1 [file cancers-15-00199-s001.zip › cancers-2049110-Supplementary Figure S1 PRISMA 2009 flow diagram.pdf]
