# Supplementary material for: Margin Free Resection Achieves Excellent Long Term Outcomes in Parathyroid Cancer
Source: Cancers (Basel). 2022 Dec 29;15(1):199. doi: 10.3390/cancers15010199 (PMC9818355; doi:10.3390/cancers15010199)
Supplement: Supplementary file 1 [file cancers-2049110-Supplementary Table S3.pdf › cancers-2049110-Supplementary Table S3.pdf]

Supplementary Table S3: Outcome in register studies of parathyroid cancer											
Year	Lead author	ref	type	n	Follow-up<br>- at 5 years<br>- median	OS<br>5-year	RFS<br>5-year	DSS<br>5-year	OS<br>10-year[a]	RFS<br>10-year[b]	DSS<br>10-year
1999	Hundahl	[46]	NCBD<br>1985-1995	286	5y-FU: 134	**115/134<br>85.5%**			49.1%		
2011	Schaapveld	[45]	Netherlands<br>1989-2003	41	5y-FU=41	**33/41<br>80.4%**	32/41<br>79.0%	34/41<br>82.5%	28/41<br>68%	29/41<br>70.8%	32/41<br>78.9%
2014	Hsu	[47]	SEER<br>1988-2014	405	5y-FU: n.a.<br>med: 5.7y	82.5%		94.1%	65.4%		89.9%
2014	Sadler	[10]	NCDB<br>1998-2011	1022	5y-FU: 528<br>med: 6.4y	**428/528<br>81.1%**	-	-	-	-	-
2014	Villar-del-Moral	[48]	Spanish Ass. Surgery	62	5y-FU: n.a.<br>med: 4.5y	(79.7%)	45/59<br>76.3%[c]	54/59<br>92.2%	n.a.	69.1%	69.1%
2015	Asare	[49]	NCBD<br>1985-2006	733	5y-FU =733	82.3%	-	-	66%	-	-
2016	Christakis	[50]	MDACC<br>1980-2015	31	5y FU=31	26/31<br>82.6%	18/31<br>59.6%	n.a	24/31<br>76.3%	n.a	n.a
2016	Young	[51]	California Cancer Registry	136	5y FU=136	**118/136<br>86.9%**	-	-	98/136<br>72%	-	-
2017	Ryhänen	[8]	Finland<br>2000-2011	32	5y-FU: 23<br>meanFU:6.7y	**20/23<br>90.6%**	17/23<br>73.9%	20/23<br>87.0%	13/18<br>71.9%	12/18<br>66.7%	14/18<br>77.8%
2017	Wang	[52]	Chinese database	234	5y FU=215	-	-	178/215<br>83%	-	-	144/215<br>67%
2017	Silva-Figueroa	[38]	MDACC<br>1980-2016	68	5y<br>FU= 68	54/68<br>80%	42/68<br>62%	-	46/68<br>68%	35/68<br>51%	-
2019	Asare	[53]	MDACC<br>1980-2017	75	5y-FU<br>=75	**59/75<br>78%**	-	-	46/75<br>61%	-	-
2021	Kong	[54]	Korea<br>2002-2017	255	5y-FU: n.a.<br>med: 5.7y	**221/255<br>86.5%**	-	-	(72.9%)	-	-
2022	Ullah	[11]	SEER<br>1975-2016	609	5y-FU: 511	**428/511<br>83.8%**	-	-	n.a.	-	-
2022	Qian	[55]	SEER<br>2001-2018	604	5y-FU: n.a.<br>med: 6.3y	n.a.			n.a.		
2022	Zhang	[13]	SEER<br>2000-2018	590	5y-FU: n.a.	477/590<br>80.8%		93.6%	67.1%		92.1%
	**Register data**		**Only bold studies counted to avoid duplication from SEER and NCBD register**			**1422/1703<br>83.5%**	**59.6-79%**	**82.5-94%**	**49-72.9%**	**51-70%**	**67-92%**
**2022**	**Schulte**		**single**	**25**	**5y-FU:24**	**23/24<br>95.8%**	**19/23<br>82.6%**	**19/19<br>100%**	**16/19<br>84.2%**	**15/19<br>79.0%**	**23/23<br>100%**

a Denominator: n patients who died of any cause or have a follow-up of 10 years; numerator: n patients alive patients at FU of at least 120 months
b Denominator: n patients who died of any cause or have a follow-up of 10 years; numerator: n patients D/other or alive at FU of at least 120 months without recurrence
c Estimated from Kaplan-Meier graph in Fig 1, also see "outcome data"
